# The Effects of Short-Term Heavy Load Squat vs. Moderate Load Olympic Weightlifting Training on Maximal Strength and Force–Velocity Profile in Young Female Handball Players

**DOI:** 10.3390/jfmk10020187

**Published:** 2025-05-23

**Authors:** Gergely Pálinkás, Csaba Ökrös, Zsófia Tróznai, Katinka Utczás, Leonidas Petridis

**Affiliations:** 1 Research Center for Sport Physiology, Hungarian University of Sport Science, 1123 Budapest, Hungary; palinkas.gergely@tf.hu (G.P.);; 2 Sports Games Department, Hungarian University of Sport Science, 1123 Budapest, Hungary

**Keywords:** FV profile, power training, intervention, strength training

## Abstract

**Objectives**: This study examined changes in maximal strength and the force–velocity (FV) profile in young female handball players following an in-season heavy load squat or a moderate load Olympic weightlifting short-term training intervention. **Methods**: A total of 27 active young female handball players (age: 17.6 ± 0.98 years; height: 168.9 ± 5.1 cm; weight: 64.9 ± 10.6 kg) participated. 5RM back squats and unloaded and loaded countermovement jumps were assessed to establish the FV profile. Participants were divided into the following groups: the control group (CG; *n* = 8) followed its usual strength training including bodyweight exercises, the heavy load squat group (SQUAT; *n* = 7) performed heavy load back squats, and the Olympic weightlifting group (OWG; *n* = 12) used moderate load weightlifting derivates. **Results**: Absolute and relative 5RM back squat and vertical jump height increased in both intervention groups (5RM from 56.8 ± 5.5 to 62.2 ± 5.3 kg, *p* < 0.05 and CMJ height from 26.7 ± 4.7 to 28.4 ± 4.9 cm, *p* < 0.05 for SQUAT; 5RM from 56.6 ± 6.8 to 66.1 ± 6.9 kg, *p* < 0.05 and CMJ height from 26.9 ± 3.0 to 28.3 ± 3.2 cm, *p* < 0.05 for OWG), but not in the CG (5RM from 63.8 ± 12.5 to 63.4 ± 12.9 kg, CMJ height from 28.4 ± 2.2 to 27.7 ± 2.9 cm). The FV profile did not change significantly in either group. The theoretical maximal force remained almost unchanged in the SQUAT and OWG (+2% each), while only the OWG could slightly decrease FV imbalance (−14%). **Conclusions**: Both strength and Olympic weightlifting short-term training could improve strength and explosive performance, but without affecting the FV profile in young, less skilled female handball players. Olympic weightlifting may offer a slight advantage due to its potential to improve power production while optimizing FV imbalance.

## 1. Introduction

Handball is a sport involving intermittent high intensity movements, accelerations, decelerations, jumps, and multidirectional changes of direction, often combined with physical contact [1]. Given the importance of explosive strength and power in handball, assessment and training of these attributes has gained increased attention from coaches and sport practitioners over the last years. Strength is the ability to exert force against an external resistance [2], while power is the amount of force produced at a given velocity [3].

Various intensities or the number of sets and repetitions provide different stimuli in strength training. Typically, high-resistance exercises (e.g., squats, dead lift) train maximal strength, while low-resistance exercises benefit the rate of force development, developing explosive strength [4]. Nevertheless, a combination of loading strategies have been suggested to optimize gains in strength and power [4]. The force–velocity (FV) profiling introduced by Samozino et al. [5] has been used as a diagnostic tool in optimizing strength and power training according to athletes’ individual needs. Accordingly, strength training with heavy loads targets the force end of the FV curve, whereas more explosive movements with light loads targets the velocity end of the FV curve [6]. It has been also suggested that the FV profile reflects the strength profile of various sports [7] as well as physiological adaptation to strength training. Jiménez-Reyes et al. [8,9] have demonstrated that strength training based on an individual FV profile can effectively develop explosive strength, resulting in significant improvements in jumping and sprinting ability.

Although strength and conditioning are often implemented in the training programs of handball players, there still seems to be uncertainty regarding the most effective method to increase the strength and power capabilities of athletes. It is generally accepted that traditional strength training with basic movements like squats, deadlifts, and presses has a positive effect on explosiveness [10], yet training methods in handball are often based on empirical knowledge rather than evidence-based research [1]. In addition, time efficiency is an important consideration in strength and conditioning training, given the large total training load in handball due to multiple training demands (e.g., physical, technical, or tactical training) [1]. While previous research has mainly compared the effects of heavy vs. light resistance training [11], Olympic weightlifting exercises have appeared as a favorable option providing significant benefits in power training [12]. Evidence in male handball players suggests that training programs using Olympic weightlifting movements with high speed can effectively improve explosiveness [11,13]. These results can be attributed to the distinct characteristic of Olympic weightlifting exercises to utilize both force and velocity against moderate loads with maximal acceleration [14]. Another important aspect is the synchronous triple extension of hip, knee, and ankle joints, which is quite common in a plethora of basic movements (e.g., jumping, sprinting). Therefore, it is expected that, due to the similarities in movement patterns, improvement in Olympic weightlifting exercises may be transferred to higher athletic performance [4]. Yet, the connection of Olympic weightlifting training with changes in the FV profile, particularly in female athletes, is still unclear. Most studies in handball-specific research have involved male athletes, while significantly less data is available regarding strength and power development in female handball players. In a study with elite handball and volleyball players, a large force deficit was found in the vast majority of the players, indicating a substantial need for maximal strength development [15]. It has been suggested that the strength and power training of young, less skilled athletes should first emphasize maximal strength training [4]. However, it is unknown whether moderate-load Olympic weightlifting exercises can offer additional benefits in reactive strength and power compared to a more traditional, maximal strength training program for young female handball players with limited experience in strength training.

Therefore, the purpose of this study was to investigate changes in maximal strength and vertical FV profiles in young female handball players following a short-term heavy load maximal strength training or a moderate load Olympic weightlifting-based intervention. We expected that weightlifting training would induce a larger increase in power output compared to strength training, and it was hypothesized that the latter would shift the FV profile towards force dominance and the former towards velocity dominance.

## 2. Materials and Methods

### 2.1. Study Design

**Figure 1:**
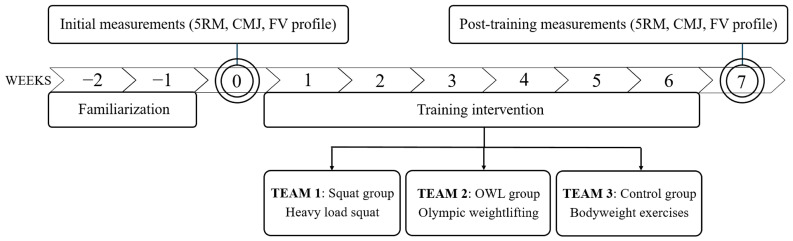
Flowchart of the study design. 5RM = five-repetition maximum in back squat, CMJ = countermovement jump; FV = force–velocity; Squat group = performed heavy load back squat training; OWG = performed moderate load Olympic weightlifting exercises and their derivatives; Control group = performed bodyweight and light resistance exercises.

All participants underwent a testing session that consisted of a basic anthropometric assessment, maximal strength testing, and FV profile testing. All measurements were conducted in the training facilities of each team in a standardized sequence. Before testing, a two-week familiarization period with weightlifting exercises was provided. From the subsequent week after pre-training assessments, a six-week training intervention was applied, followed by the post-training assessments. The training intervention consisted of two sessions per week, each session lasting approximately 60 min. Irrespective of their type of training intervention, all players followed an almost identical handball specific training (5 × 120 min/week) and played a competitive match on weekends. Participants were divided into three groups based on the training program they had to follow: the Control Group (CG) performed bodyweight exercises, the Squat Training Group (SQUAT) performed heavy load back squat training, and the Olympic Weightlifting Training Group (OWG) included moderate load Olympic weightlifting exercises and their derivatives. The overall structure of the study is represented in Figure 1.

### 2.2. Participants

A total of 40 female handball players from three different handball clubs were selected to participate in the study. The allocation of each team to one of the study groups was randomized; accordingly, the players within the same team followed the same training corresponding to their allocation. Inclusion criteria included regular attendance of team training sessions, at least three years of handball-specific training experience, a valid license to participate in official regional or national competitions, and a lack of any injury at least 3 months before the measurements. For the post-training assessments, a maximum of one missed training session was allowed. Due to illness or injury during the intervention period or inadequate training attendance, 13 players were excluded, and finally, 27 participants met all inclusion criteria. The basic data of the participants can be found in Table 1. Before the measurements, all participants received written and verbal information about the measurements and the training protocols, then written consent was obtained from their parents or legal guardians to participate in the study. The study was approved by the University’s Research Ethics Committee (approval number: TE-KEB/NO34/2019).

### 2.3. Measurement Protocol

Body height was measured with a stadiometer to the nearest 0.1 cm, and body mass (BM) was measured with a digital scale (Seca 888, seca GmbH & Co. KG., Hamburg, Germany) to the nearest 0.1 kg. Countermovement jumps (CMJ) and FV profiles were assessed on a force platform (Model FP4, HUR Labs Oy, Tampere, Finland) at a 1000 Hz sampling rate. Prior to testing, participants performed a standardized warm up protocol consisting of dynamic stretching exercises, squats, lunges, and three submaximal jumps. Countermovement jumps were performed with hands on hips to exclude additional momentum of the upper limbs. Participants performed three maximal jumps with a rest of one minute in-between. Jump height (cm) and takeoff velocity (m/s) based on the impulse–momentum method, maximal force (N), and maximal power (W) were extracted from the force plate for the unloaded jumps. To assess the FV profile, three additional vertical jumps with external loading were performed as suggested previously [7]. A barbell placed behind the neck in the back rack position provided external loading with resistances corresponding to 25%, 50%, and 75% of body mass. Participants had to perform two vertical jumps at each load condition with two minutes of rest in-between; a minimum of a 10-cm jump against each external load was required. Mean force during the push-off phase (when ground reaction force becomes greater than bodyweight to the instant of takeoff) was registered at every loaded condition. Push off distance (*hpo*) was defined as the vertical displacement of the centre of mass moved from the lowest point of the countermovement to the instant of takeoff, and it was extracted from the vertical displacement–time curve of the force platform. Average speed was calculated from jump height according to Samozino et al. [5] and maximal power from maximal force and maximal velocity according to Vandewalle et al. [16]. The maximal theoretical Force (*F*_0_—N), maximal theoretical velocity (*v*_0_—m/s), maximal theoretical power (*Pmax*—W), the slope of the measured and the optimal FV profile (calculated from *Pmax* and *hpo*), and the FV imbalance were used in the statistical analysis.

Maximal dynamic strength was assessed with a Five-Repetition Maximum protocol (5RM) in back squats. To reach 5RM, participants performed five sets of five repetitions with back squats in the high bar position. Given that participants were not proficient in 5RM testing, we used the rate of perceived exertion (RPE) instead of a more common percentage-based ascending to define progression in resistance loading based on previous reports that related RPE scale with training intensity in adolescent athletes [17]. During the five sets, the athletes had to reach 5, 7, 8, 9, and then 10 out of a scale of 10 in RPE. Athletes were informed of the features of the 10-grade scale (e.g., heart rate, breathing, muscle fatigue), where 1 corresponds to rest and 10 to maximal effort. 5RM was defined as the last load at which participants could perform five repetitions according to the technique requirements. The technique requirements were stability of the torso and the hips descending below the knees. If a participant could not perform five repetitions with proper technique (or at all) the last valid step was accepted as the 5RM.

### 2.4. Training Protocol

The control group continued its usual strength and conditioning training regimen, which primarily consisted of light bodyweight strengthening. Resistance training in this group was implemented using resistance bands and bodyweight exercises performed for 8–10 repetitions per set.

The SQUAT group performed a linearly periodized back squat training program with maximal speed. The training load was defined from the 1RM (66.8 ± 6.5 kg), which was estimated from the 5RM deep squat obtained during the initial assessment [18]. The initial load corresponded to 80% of 1RM, and then relative intensity increased by 2% every week, reaching 92% at the last week, while the number of sets and repetitions remained constant (5 sets × 3 repetitions).

The OWG adopted an Olympic weightlifting-based training program under the supervision of a weightlifting coach. The training program for this group was built upon simplified variations of the snatch and clean & jerk movements like hang power snatch, power cleans, and push presses. External resistance for the derivative exercises (e.g., squats and deadlifts) was determined based on the loads used in the weightlifting exercises. Considering that the participants had no prior technical experience with Olympic weightlifting, the relative intensity of these exercises was low (~60–65% of their 1RM back squat). The number of sets and repetitions remained constant (3 sets of 2 repetitions for both the snatch and clean & jerk derivates).

### 2.5. Statistical Analysis

For the statistical analysis, we used IBM SPSS Statistics (V29.0.1.0) software. The Shapiro–Wilk test for normality revealed a normal distribution across all examined variables. The effect of the training programs across the three groups was analyzed using a factorial repeated-measures ANOVA with the group as the independent factor. Within-group pre- and post-training comparisons were conducted using repeated measures ANOVA for each group separately. To control for family-wise errors, Bonferroni correction was applied. Partial eta squared (η_p_^2^) was used to assess the effect size, where 0.02 was low, 0.06 moderate, and 0.14 large. The results are presented as mean ± standard deviation. Statistical significance was set at *p* < 0.05.

## 3. Results

The results of the repeated measures ANOVA for the 5RM squat and the CMJ are summarized in Table 2, and the descriptive results before and after the training intervention are in Table 3. The results indicated significant main effects of time and significant interaction between time and group with no significant main effects for groups (Table 2). The absolute and relative 5RM squat performance, representing maximal strength, improved in both strength training and Olympic weightlifting groups with large effect sizes, but not in the control group (Table 3). A similar trend was observed for vertical jump performance.

Repeated measures ANOVA testing for the FV profile measurements revealed no significant results either for the main effects of time or group or for the interaction between time and group (Table 4). Force–velocity variables included the theoretical maximal force (*F*_0_), the theoretical maximal velocity (*v*_0_), the theoretical maximal power (*Pmax*), and the change in the measured FV profile slope.

The changes of the FV profile for each group are presented in Table 5. The theoretical maximum force (*F*_0_) decreased in the control group, while it slightly (non-significantly) increased in the other two groups. The theoretical maximum velocity (*v*_0_) remained almost unchanged in the weightlifting group and the control group, but it slightly decreased in the maximal strength group. The theoretical maximal power (*Pmax*) did not change in the weightlifting group, while it declined (but not significantly) in the other two groups. FV imbalance also changed differently in the examined groups: it decreased in the OWG, it increased in the CG, and it remained the same in the SQUAT.

## 4. Discussion

The aim of this study was to examine the effects of a short-term heavy load squat versus moderate load Olympic weightlifting training intervention on the strength capacity and FV profiles of young female handball players. The results revealed significant improvements in maximal and reactive strength in both intervention groups, but with no concomitant changes in the FV profile metrics. No statistically significant difference was observed between the two types of strength training. The control group did not change either in maximal or in reactive strength. These results confirm the well-known effects of resistance training on reactive force and power [11,19,20]; however, the effects on the FV profile remain unclear.

Maximal strength training has been suggested to provide superior benefits in 1RM and work economy over more conventional training modalities (e.g., low resistance) [21]. Other, less common training methods (such as trampoline training) may yield protective effects on leg stiffness, offering alternative methods in strength training [22]. Although in less skilled and young athletes it is recommended to prioritize maximal strength in strength training [4], it is unclear whether Olympic weightlifting exercises performed at moderate load and maximal velocity could offer additional (or equal) benefits in maximal strength and FV profile.

The 5RM squat test represented the maximal strength capacities of the athletes, but, contrary to our expectations, the athletes training at a higher load (strength training group) exhibited smaller improvements compared to those engaged in weightlifting training. This contradicts previous studies suggesting that loading is a stronger influencing factor in maximal strength development than volume [23]. One possible explanation is the greater engagement of core musculature in the OWG due to variations in bar placement. While the strength training group performed back squats with the bar always placed on the upper back, the weightlifting group also performed front squats and overhead squats. These variations in squat techniques result in different muscle activation patterns, yielding similar strength development while reducing joint loading [24]. The control group also included squats in their training program, but mostly with dumbbells using higher repetitions against light loads, leading to reduced core engagement.

Despite the improvement in jump height during the unloaded countermovement jumps, the FV profile did not change in any of the examined groups. We expected a clear shift towards force dominance for the strength training group, yet changes in the theoretical maximal force were similar for both intervention groups. This indicates a weak connection of the FV profile changes with the type of strength training in young female athletes. This idea is supported also by the large inter-individual variability in the FV changes after an acute training load [25]. Irrespective of the type of strength training, the FV profile moved either to the force or the velocity dominance direction, indicating a less straightforward interpretation of the effects of strength training on the FV profile. Players had limited experience in strength training, and it is possible that for novice athletes, strength training of any kind can result in similar changes in their FV profile. These findings contradict previous studies including well-trained male athletes which suggested that the FV profile responds differently according to the specific training intervention [9].

Interestingly, the theoretical maximal power decreased in the strength training (and the control) group, but it remained relatively unchanged in the weightlifting group. In other words, both intervention groups yielded similar changes in maximal force, but only the weightlifting group could maintain maximal power. This indicates that weightlifting training appears to be somewhat more optimal for improvement in power output compared with the strength training group. At the same time, FV imbalance decreased only in the OWG (approaching optimal imbalance), although it is important to note that before and after the training intervention, the imbalance remained within the low-deficit range [9]. It seems, therefore, that a short-term maximal strength or Olympic weightlifting training program could not significantly affect the FV imbalance in young female handball players. It is possible that longer training periods may be necessary to observe significant changes in the FV profile. However, it should be noted that, during the assignment into the strength or Olympic weightlifting training groups, we did not consider the initial FV profile of the players, which may yield more favorable effects on the FV profile as reported previously [9]. Nevertheless, the initial goal was to examine the main effects of strength or Olympic weightlifting training of the FV profile irrespective of the individual profile of the players.

Our results are limited by the small sample size, which affects statistical analysis. While initially, all three groups consisted of 13–14 players, injuries or incomplete training attendance reduced the number of participants in each group. While Olympic weightlifting exercises were intended to be performed with maximal velocity, the realization of this during the training sessions was limited by their technical proficiency. Most likely, the athletes in the Olympic weightlifting group could not achieve maximal velocity, affecting the effects of training. Further, menstrual cycles were not controlled for, and neither was contraceptive use, dieting, or the use of any dietary supplements. Hormonal changes during the different phases of the menstrual cycle or in response to contraceptive use may influence training adaptation and accordingly performance in testing protocols.

The findings highlight the beneficial effects of both heavy load strength training and Olympic weightlifting training at moderate loads on maximal and reactive force. In addition, while the theoretical maximal force of the FV profile exhibited comparable (slight) increases in both intervention groups, only the weightlifting group could maintain maximal power output and moderately reduce the FV imbalance. Overall, it seems that, in young female athletes with limited experience in strength training, improvements in maximal and reactive strength can be achieved during the competitive season through resistance training, with both examined training modalities yielding comparable benefits. Meanwhile, the absence of any improvement in the control group, which only performed bodyweight exercises and light conditioning, emphasizes the need to implement resistance training. While maximal and reactive strength improved, the FV profile did not change, likely indicating that the initial individual FV profile may be more important to induce favorable changes than the type of training and should be considered when planning strength training programs.

## Figures and Tables

**Table 1:** Descriptive data of the participants.

	Control Group	Squat Group	Olympic Weightlifting Group
*n*	8	7	12
Age (years)	17.7 ± 0.7	18.0 ± 1.4	17.4 ± 0.9
Body Height (cm)	171.1 ± 3.4	168.9 ± 4.9	167.3 ± 5.9
Body Mass (kg)	65.8 ± 11.2	64.8 ± 6.6	64.3 ± 12.8

**Table 2:** Results of the repeated measures ANOVA for the five-repetition maximum back squat and countermovement jump assessments.

	Time	Group	Interaction
5RM Back Squat	F(1,24) = 45.2; *p* < 0.001; η_p_^2^ = 0.65	F(2,24) = 0.44; *p* > 0.05; η_p_^2^ = 0.04	F(1,24) = 17.2; *p* < 0.001; η_p_^2^ = 0.59
Relative 5RM Back Squat	F(1,24) = 43.04; *p* < 0.01; η_p_^2^ = 0.64	F(2,24) = 0.32; *p* > 0.05; η_p_^2^ = 0.03	F(2,24) = 16.04; *p* < 0.01; η_p_^2^ = 0.57
CMJ Height	F(1,24) = 9.68; *p* < 0.01; η_p_^2^ = 0.29	F(2,24) = 0.05; *p* > 0.05; η_p_^2^ = 0.00	F(2,24) = 8.18; *p* < 0.01; η_p_^2^ = 0.41
Takeoff Velocity	F(1,24) = 8.55; *p* < 0.01; η_p_^2^ = 0.26	F(2,24) = 0.07; *p* > 0.05; η_p_^2^ = 0.01	F(2,24) = 8.30; *p* < 0.01; η_p_^2^ = 0.41

5RM = five-repetition maximum; CMJ = Countermovement Jump.

**Table 3:** Results of the five-repetition maximum back squat and countermovement jump for the three groups before and after the training intervention (mean ± SD).

	Group	Before	After	Change (%)	η_p_^2^
5RM Back Squat (kg)	OWG	56.6 ± 6.8	66.1 ± 6.9	17% *	0.83
	SQUAT	56.8 ± 5.5	62.2 ± 5.3	10% *	0.88
	CG	63.8 ± 12.5	63.4 ± 12.9	−1%	0.01
Rel. 5RM Back Squat (kg/BM)	OWG	0.9 ± 0.18	1.0 ± 0.15	17% *	0.82
	SQUAT	0.9 ± 0.11	1.0 ± 0.10	10% *	0.91
	CG	1.0 ± 0.18	1.0 ± 0.15	0%	0.01
CMJ Height (cm)	OWG	26.9 ± 3.0	28.3 ± 3.2	5% *	0.71
	SQUAT	26.7 ± 4.7	28.4 ± 4.9	7% *	0.82
	CG	28.4 ± 2.2	27.7 ± 2.9	−2%	0.12
Takeoff Velocity (m/s)	OWG	2.3 ± 0.13	2.4 ± 0.13	3% *	0.71
	SQUAT	2.3 ± 0.21	2.4 ± 0.21	3% *	0.80
	CG	2.4 ± 0.09	2.3 ± 0.12	−1%	0.13

OWG = Olympic weightlifting group; SQUAT = heavy load squat training group; CG = control group; 5RM = five-repetition maximum; CMJ = countermovement jump; * = significant change between pre- and post-training intervention.

**Table 4:** Results of the repeated measures ANOVA for the force–velocity profile.

	Time	Group	Interaction
*F*_0_	F(1,24) = 0.26; *p* > 0.05; η_p_^2^ = 0.01	F(2,24) = 0.55; *p* > 0.05; η_p_^2^ = 0.04	F(2,24) = 1.44; *p* > 0.05; η_p_^2^ = 0.11
*v*_0_	F(1,24) = 1.07; *p* > 0.05; η_p_^2^ = 0.04	F(2,24) = 0.27; *p* > 0.05; η_p_^2^ = 0.02	F(2,24) = 0.32; *p* > 0.05; η_p_^2^ = 0.03
*Pmax*	F(1,24) = 1.11; *p* > 0.05; η_p_^2^ = 0.04	F(2,24) = 0.37; *p* > 0.05; η_p_^2^ = 0.03	F(2,24) = 0.752; *p* > 0.05; η_p_^2^ = 0.06
FV slope	F(1,24) = 0.02; *p* > 0.05; η_p_^2^ = 0.00	F(2,24) = 0.06; *p* > 0.05; η_p_^2^ = 0.00	F(2,24) = 0.51; *p* > 0.05; η_p_^2^ = 0.04

*F*_0_ = theoretical maximum force (N); *v*_0_ = theoretical maximal velocity (m/s); *Pmax* = theoretical maximal power; FV slope: slope of the force–velocity relationship

**Table 5:** Results of the force–velocity profile for the three groups before and after the training intervention (mean ± SD).

	Group	Before	After	Change (%)	η_p_^2^
*F*_0_ (N/kg)	OWG	28.8 ± 3.1	29.4 ± 3.2	2%	0.03
	SQUAT	27.8 ± 3.7	28.4 ± 4.4	2%	0.05
	CG	29.0 ± 3.1	26.4 ± 5.8	−9%	0.15
*v*_0_ (m/s)	OWG	3.0 ± 0.7	3.0 ± 0.6	0%	0.00
	SQUAT	3.1 ± 1.7	3.0 ± 0.6	−4%	0.08
	CG	3.1 ± 1.2	3.1 ± 1.1	0%	0.06
*Pmax* (W)	OWG	21.3 ± 3.8	21.7 ± 3.1	2%	0.01
	SQUAT	22.2 ± 7.8	20.9 ± 4.0	−6%	0.04
	CG	21.8 ± 5.9	17.7 ± 11.3	−19%	0.11
FV slope (Ns/m/kg)	OWG	0.68 ± 0.22	0.70 ± 0.19	12%	0.01
	SQUAT	0.67 ± 0.27	0.71 ± 0.22	26%	0.03
	CG	0.71 ± 0.25	0.62 ± 0.28	−8%	0.06
FVimb (%)	OWG	35 ± 16	31 ± 23	−14%	0.04
	SQUAT	33 ± 27	33 ± 14	0%	0.05
	CG	31 ± 23	38 ± 28	7%	0.00

OWG = Olympic weightlifting group; SQUAT = heavy load squat training group; CG = control group; *F*_0_ = theoretical maximum force (N); *v*_0_ = theoretical maximal velocity (m/s); *Pmax* = theoretical maximal power; FV slope: slope of the force–velocity relationship; FVimb = imbalance of force–velocity profile.

## Data Availability

Research data are available upon reasonable request from the corresponding author.

## Abbreviations

The following abbreviations are used in this manuscript:

FV profile	Force–velocity profile
*F*_0_	Theoretical maximum force
*v*_0_	Theoretical maximum velocity
*Pmax*	Theoretical maximum power
SQUAT	Heavy load squat training group
OWG	Olympic weightlifting group
CG	Control group
